# Fe_3_O_4_-Au Core-Shell Nanoparticles as a Multimodal Platform for In Vivo Imaging and Focused Photothermal Therapy

**DOI:** 10.3390/pharmaceutics13030416

**Published:** 2021-03-20

**Authors:** Carlos Caro, Francisco Gámez, Pedro Quaresma, Jose María Páez-Muñoz, Alejandro Domínguez, John R. Pearson, Manuel Pernía Leal, Ana M. Beltrán, Yilian Fernandez-Afonso, Jesús M. De la Fuente, Ricardo Franco, Eulália Pereira, Maria Luisa García-Martín

**Affiliations:** 1 BIONAND—Centro Andaluz de Nanomedicina y Biotecnología (Junta de Andalucía-Universidad de Málaga), C/Severo Ochoa, 35, 29590 Málaga, Spain; ccaro@bionand.es (C.C.); jpaez@bionand.es (J.M.P.-M.); adominguez@bionand.es (A.D.); jrpearson@bionand.es (J.R.P.); 2 Departamento de Química Física, Universidad de Granada, Avenida de la Fuente Nueva S/N, 18071 Granada, Spain; fgammar@gmail.com; 3 REQUIMTE/LAQV, Departamento de Química e Bioquímica, Faculdade de Ciências da Universidade do Porto, 4169-007 Porto, Portugal; pedro.cq1@gmail.com; 4 Departamento de Química Orgánica y Farmacéutica, Facultad de Farmacia, Universidad de Sevilla, 41012 Seville, Spain; mpernia@us.es; 5 Departamento de Ingeniería y Ciencia de los Materiales y del Transporte, Escuela Politécnica Superior, Universidad de Sevilla, Virgen de África 7, 41011 Sevilla, Spain; abeltran3@us.es; 6 Instituto de Nanociencia y Materiales de Aragón (INMA), CSIC-Universidad de Zaragoza, 50009 Zaragoza, Spain; yfdezafonso@gmail.com (Y.F.-A.); jmfuente@unizar.es (J.M.D.l.F.); 7 Biomedical Research Networking Center in Bioengineering, Biomaterials &Nanomedicine (CIBER-BBN), 28029 Madrid, Spain; 8 UCIBIO, REQUIMTE, Departamento de Química, Faculdade de Ciências e Tecnologia, Universidade NOVA de Lisboa, 2829-516 Caparica, Portugal; rft@fct.unl.pt

**Keywords:** imaging-guided therapy, multimodal imaging, contrast agent, MRI, CT

## Abstract

In this study, we report the synthesis of gold-coated iron oxide nanoparticles capped with polyvinylpyrrolidone (Fe@Au NPs). The as-synthesized nanoparticles (NPs) exhibited good stability in aqueous media and excellent features as contrast agents (CA) for both magnetic resonance imaging (MRI) and X-ray computed tomography (CT). Additionally, due to the presence of the local surface plasmon resonances of gold, the NPs showed exploitable “light-to-heat” conversion ability in the near-infrared (NIR) region, a key attribute for effective photothermal therapies (PTT). In vitro experiments revealed biocompatibility as well as excellent efficiency in killing glioblastoma cells via PTT. The in vivo nontoxicity of the NPs was demonstrated using zebrafish embryos as an intermediate step between cells and rodent models. To warrant that an effective therapeutic dose was achieved inside the tumor, both intratumoral and intravenous routes were screened in rodent models by MRI and CT. The pharmacokinetics and biodistribution confirmed the multimodal imaging CA capabilities of the Fe@AuNPs and revealed constraints of the intravenous route for tumor targeting, dictating intratumoral administration for therapeutic applications. Finally, Fe@Au NPs were successfully used for an in vivo proof of concept of imaging-guided focused PTT against glioblastoma multiforme in a mouse model.

## 1. Introduction

Despite being the most frequent and aggressive primary malignant brain tumor in adults, few therapeutic advances have been achieved against glioblastoma multiforme (GBM). The median survival of GBM patients after diagnosis is only about 15 months, with a five-year survival rate of 3–5% [1,2]. Therefore, the development of new diagnosis methodologies and more effective therapies is an urgent challenge [3].

The most widely used diagnostic tools for GBM are magnetic resonance imaging (MRI) and X-ray computed tomography (CT) [4,5]. MRI is one of the most powerful techniques employed in clinical diagnosis because of its noninvasive and radiochemical-free character, safety, and immediateness [6,7]. It provides excellent real-time imaging resolution of soft tissues, both in 2D and 3D, as well as important functional information [8,9,10,11]. For its part, CT is also a noninvasive technique and can generate 3D images of internal structures with high spatial and temporal resolution. CT provides exceptional visualization of bone structures, while it presents some limitations in distinguishing between tissues with similar densities, especially soft tissues [12,13]. Despite both imaging techniques exhibiting excellent features to give insight into anatomical details, some restrictions arise when the use of contrast agents (CAs) that can induce toxic or allergic reactions becomes a requirement [14,15,16,17]. A possible route to overcome some of these drawbacks can be found in nanotechnology [18]. This branch of science has been recognized as a revolutionary and promising field in the diagnosis and treatment of cancer [19,20,21,22]. For instance, superparamagnetic iron oxide nanoparticles (SPIONs) have been approved by the Food and Drug Administration (FDA) and successfully applied as CAs in bowel and liver/spleen imaging [23], while there continues to be intense research seeking new magnetic NPs with improved properties as intravenous MRI contrast agents [24]. Gold NPs have also attracted considerable research interest as CAs for CT because of their high X-ray absorption coefficient [25]. Moreover, multimodal agents are an emerging research line aimed at improving diagnostic accuracy through multimodal imaging [26,27,28]. Furthermore, the standard therapy protocol for GBM, which involves surgery, radiotherapy, and chemotherapy [29], exhibits some limitations emerging mainly from the proximity of GBM to regions critical for brain functionality [30]. Nanotechnology can also improve current therapies by local drug delivery (DD), magnetic hyperthermia (MH) or photothermal therapy (PTT), and thus contribute to reduce side effects [31,32,33,34]. PTT is based on the fact that cancer cells are more sensitive to small temperature increases, which has been exploited in a conventional clinical procedure to produce controlled damage in the affected regions with minimal involvement of normal tissues by exposing target tissues to temperatures in the range of 39–45 °C [35]. In PTT, localized surface plasmon resonances (LSPRs) are externally activated to trigger a light-to-heat transduction effect that provokes damage, reparation, or modulation of biological targets [36,37]. Au NPs have recently attracted considerable attention in PTT [38,39] and also in two-photon photoluminescence (TPPL) due to their high two-photon absorption cross-section [40]. AuNPs present several advantages over organic dyes, such as the lack of photoblinking and reduced photobleaching under prolonged laser irradiation. Indeed, TPPL has been found to be enhanced by several orders of magnitude on rough metal surfaces, which is known as the lightning rod effect [41]. This effect can be explained as the combination of different LSPRs due to multiple randomly oriented nanometer-sized hemispheroids on a smooth surface. Hence, around these nanometric structures, both incoming and outgoing electric fields are amplified by the local field induced by their LSPRs [42,43]. However, the aforementioned optical properties of Au NPs present some limitations in the UV-Vis range, arising mainly from the strong phototoxicity of UV radiation and the limited tissue penetration of visible radiation [44]. Therefore, Au NPs with LSPRs in the near-IR (NIR) region, characterized by deep tissue penetration, are of utmost interest for in vivo applications [45]. A distinctive NIR wavelength region from 650–950 nm has been identified as the first biological window [46].

Multicomponent nanoparticles (MCNPs) can be described as nanoscale entities that are able to combine the properties of at least two different nanomaterials [47]. In the last few years, MCNPs have gained substantial attention because of their capabilities as multifunctional contrast and therapeutic agents in cancer theranostics [48]. On the one hand, the integration of at least two different imaging techniques (multimodal imaging) has demonstrated an improvement in diagnostic accuracy [49], as well as a chance for limiting the number of administered CAs. On the other hand, combined therapies, that is, a therapeutic approach that combines multiple treatments to create synergistic or additive effects, are currently a hot topic in cancer therapy [50]. Within this panorama, NPs based on iron oxide and gold NPs have attracted the attention of scientists because of their appealing features, such as capabilities as CAs for imaging and excellent SPR properties for biomedical applications [51,52,53].

Inspired by the above scenario, herein we report the application of surface-rough core-shell nanoparticles (Fe@Au NPs) as multimodal platforms for CT and MRI imaging and photothermal therapy. The absorbance of these NPs in the near-infrared region allows the use of radiation inside the first biological window for conventional PTT. Here, we explore the performance of a localized two-photon (TP) photothermal therapy in an in vitro 3D model of glioblastoma multiforme. Finally, imaging-guided PT therapy was also evaluated in vivo.

## 2. Materials and Methods

### 2.1. Materials

Magnetite NPs, citric acid, *N*,*N*-dimethylformamide (DMF), poly(vinylpyrrolidone) (PVP; MW: 10,000), ethyl ether, NaBH_4_, HAuCl_4_ (30% *w/w* solution in 1 M HCl), ethanol, acetone, NH_4_OH (28%), imidazole 3–4,5-dimethylthiazol-2-yl-2,5-diphenyl tetrazolium bromide (MTT), 4′,6-diamidino-2-phenylindole (DAPI), propidium iodide (PI), and phosphate buffered saline (PBS) solution were obtained from Aldrich. HCl and HNO_3_ were obtained from Panreac. Dulbecco’s Modified Eagle’s Medium (DMEM) was purchased from Gibco, Grand Island, NY, USA. Hoechst and TO-PRO-3 were purchased from Thermofisher, Waltham, MA, USA. Solutions were prepared using Millipore water (18.2 MΩ∙cm at 25 °C). All reagents were used as received without further purification.

### 2.2. Synthesis of Fe_3_O_4_@Au Core-Shell NPs

The synthesis of the core-shell NPs was carried out according to the protocol published by Quaresma et al. [54]. First, magnetic NPs were solubilized with a citric acid functionalization according to the (slightly modified) protocol of Lattuada and Hatton [55]. Then, gold seeds were deposited on the solubilized magnetic NPs and reduced with NaBH_4_. Finally, the growth of a star-shaped shell was conducted by a second reduction step using PVP and DMF [56]. Further details are provided in the Supporting Information.

### 2.3. Physicochemical Characterization

#### 2.3.1. Transmission Electron Microscopy (TEM)

Images were acquired on a FEI (Thermofischer, Waltham, MA, USA) Tecnai G2 Twin microscope, working at 100 kV. Samples were prepared as follows: 100 µL of NPs solution (concentration ≈1 g∙L^−1^ of Fe + Au) were dropped on a carbon-coated copper grid. The representative diameter was obtained as the average of 100 NPs.

#### 2.3.2. Scanning-Transmission Electron Microscopy (STEM)

STEM images were obtained on a FEI (Thermofischer, Waltham, MA, USA) TALOS F200 system with an accelerating voltage of 200 kV and equipped with a super-x energy dispersive X-ray spectrometry (EDX) system, which includes two silicon drift detectors. Compositional analyses of the samples were performed by combining high-angle annular dark-field imaging (HAADF) and EDX acquisition in STEM mode using spatial drift correction and a dwell time of 0.2 s. TEM samples were prepared by dropping 100 µL of the corresponding NPs solution at ~1 g∙L^−1^ of (Fe + Au) on a carbon-coated copper grid.

#### 2.3.3. Inductively Coupled Plasma High-Resolution Mass Spectroscopy (ICP-HRMS)

Fe and Au concentration were determined by ICP-HRMS (Perkin-Elmer NexION). NPs were digested, as previously reported by us, with aqua regia. These samples were measured in the “Servicios Centrales de Apoyo a la Investigación (SCAI)” from Malaga University.

#### 2.3.4. Fourier Transform Infrared Spectroscopy (FTIR)

Samples were measured as powder. An FTIR-4100 Jasco, equipped with an ATR accessory (MIRacle ATR, PIKE Technologies, Madison, WI, USA), was used. Spectra resolution was set as 4 cm^−1^, in the range between 4000 and 800 cm^−1^. In total, 50 scans were accumulated.

#### 2.3.5. Thermogravimetric Analysis (TGA)

Samples were measured as powder. The analysis was conducted on a Mettler-Toledo system (TGA/DSC 1), selecting the temperature range between 30 and 600 °C. Samples were measured under an N_2_ flow (50 mL∙min^−1^), and the heating rate was set as 10 °C∙min^−1^. These samples were measured in the “Servicios Centrales de Apoyo a la Investigación (SCAI)” from Malaga University.

#### 2.3.6. UV-Vis Spectroscopy

Extinction spectra were recorded on a Cary 100 (Agilent, Waltham, MA, USA) spectrometer. A quartz cuvette with a light path of 1 cm was used. NPs concentration was ~50 mg∙L^−1^ of (Fe + Au).

#### 2.3.7. Two-Photon Fluorescence Experiments

Excitation and emission spectra of the nanoparticle solutions were recorded on a Leica SP5 MP inverted confocal microscope equipped with a Spectraphysics MaiTai HP IR laser for multiphoton excitation, using a 10× HCX PL APO CS 0.70 NA objective lens. The concentration of NPs solution was ~1 g∙L^−1^ of (Fe + Au). Emissions were measured between 400 and 670 nm using multiphoton excitation from 700 to 1000 nm at 10 nm intervals. Laser power was attenuated to 1% of maximum with acquisition performed using six accumulations per line. A total of 256 × 256 pixel images were acquired for each excitation wavelength with a 1.5 × 1.5 × 3 µm voxel size.

#### 2.3.8. Dynamic Light Scattering (DLS)

Mean hydrodynamic diameter distribution and ζ-potential were analyzed on a Zetasizer Nano ZS90 (Malvern Instruments, Malvern, Worcestershire, UK). NPs were dispersed (~50 mg∙L^−1^ of (Fe + Au)) in Milli-Q water or PBS. Experiments were conducted on a ZEN0118-low disposable cuvette, setting 0.27 as refractive index with 173° backscatter (NIBS default) detection angle. Both parameters were obtained as the average of three measurements.

#### 2.3.9. Photothermal Conversion

The photothermal conversion was studied by measuring the temperature variation over time of the solution (1 g∙L^−1^) under IR irradiation. The NPs suspension was placed into a 1 cm optical path quartz cuvette and irradiated for 5 min using a laser of 1064 nm (Laser Quantum, mpc6000/Ventus 1064) while recording the suspension temperature with a fiber optic sensor (TPT-62, FISO, Technologies Inc., Quebec, QC, Canada). After that time, the laser was turned off, and the heat release was recorded for 20 min. The laser power was set to 1.22 W. The water heating contribution was identically evaluated with a NP-free sample.

#### 2.3.10. Photothermal Conversion Efficiency

The photothermal conversion efficiency (η) was evaluated according to the analytical approximation developed by Roper et al. [57,58,59], using the following equation:

(1) $$\eta =\;\frac{hS\Delta T_{max}\;-\;Q_{s}}{I\left(1\;-\;10^{-A_{1064}}\right)}$$

where *h* is the heat transfer coefficient, *S* is the surface area of the container, Δ*T_max_* is the maximum temperature variation of the system, *Q_s_* is defined as the heat associated with the NIR-light absorbance of water, *I* is the incident laser power, and *A*_1064_ is the absorbance of the system at 1064 nm. The term *hS* is calculated by the linear fit of the plot of ln[(*T* − *T*_0_)/(*T*_∞_ − *T*_0_)] against *t*. Further details can be found in the original reference.

#### 2.3.11. Hyperthermia Calculations

The temperature around an isolated nanoparticle was estimated by numerically solving the Fourier law with the finite elements method with the help of the FEMM software (https://groups.io/g/femm/, accessed on September 2020). All three layers (magnetic core, gold shell, and polymer coating) were employed to analyze the temperature around the absorbing NP to a continuum water medium. The emitting power was evaluated from the laser power density and the absorption cross-section derived from Mie theory for the stratified NP [60]. It was assumed that radiation is effectively absorbed within the gold layer. The temperature at the surface of the nanoparticle, *T*_s,_ is a very good approximation of the terminal temperature (*T*_∞_, the temperature at a time *t* = ∞) of the surrounding medium. We also fitted the experimental hyperthermia curves to the analytical solution of the Fourier equation accounting for the continuous heating of a homogeneous medium by a local heat source. From the experimental photothermal conversion data, the optical specific absorption rate (oSAR in W∙g^−1^) was evaluated. oSAR is defined here as the power dissipation per unit mass of NP (*m_NP_*):

(2) $$oSAR=\frac{CV}{m_{NP}}\times \frac{dT}{dt}$$

where *dT*/*dt* is the temperature increase as a function of time, and it was calculated at a time *t* = 2 min. *C* is the specific heat capacity of the sample, and *V* is the total volume.

### 2.4. In Vitro Experiments

#### 2.4.1. Cell Culture

HFF-1 human fibroblasts and C6 rat glioma cells were cultured at 37 °C in an incubator with a humidified atmosphere containing 5% CO_2_. The growth medium was DMEM supplemented with 2 mM of L-glutamine, 10% of fetal bovine serum (FBS) and 1% of penicillin/streptomycin.

#### 2.4.2. Cell Morphology Studies

Cell morphology was evaluated on a Perkin Elmer Operetta High Content Imaging System. Experimental details are provided in the Supporting Information.

#### 2.4.3. Cytotoxicity Assays

Cytotoxicity was assessed using the MTT assay, which is described in detail in the Supporting Information, in both HFF-1 and C6 cells.

#### 2.4.4. In Vitro Transverse Relaxivities (r_2_)

Transverse relaxivities were measured at 1.44 T and 9.4 T, using Fe concentrations ranging between 0.5 and 0.05 mM in physiological conditions. On the one hand, transverse relaxation times (T_2_) at 1.44 T were obtained on a Bruker Minispec system using the Carl-Purcell-Meiboom-Gill (CPMG) spectroscopic sequence. On the other hand, T_2_ at 9.4 T were measured on a Bruker Biospec MRI system equipped with 400 mT∙m^−1^ field gradients and a 40 mm quadrature bird-cage resonator at 298 K. In this magnetic field, a 64-echo CPMG image sequence, with echo-time (TE) values from 7.5 to 640 ms, was used. Relaxivities at both magnetic fields were calculated from the slope of the linear fit of the relaxation rate (1/T_2_) versus Fe concentration. See further details in the Supporting Information.

#### 2.4.5. In Vitro Computed Tomography (CT)

The CT images (*n* = 1000) were acquired on a Bruker Albira small animal CT system, with an X-ray focal spot size (nominal) of 35 μm and setting the energy to 45 kVp at 400 µA. Resulting HU were adjusted to see differences between soft tissues (leaving the bones overexposed). X-ray attenuation coefficients in Hounsfield units (HU) were plotted against gold concentrations to calculate the slope.

#### 2.4.6. In Vitro Two-Photon Photodynamic Therapy

Experiments were performed using a Leica SP5 MP inverted confocal microscope equipped with a SpectraphysicsMaiTai HP IR laser for multiphoton excitation, using a 20× HCX PL APO CS 0.70 NA objective lens. Cells were grown on 35 mm glass-bottomed dishes (Ibidi) and maintained at 37 °C under an atmosphere of CO_2_ (5%) in a microscope incubator throughout the experiment. LIVE/DEAD staining was carried out by adding 1 µg∙mL^−1^ Hoechst and 3 µM propidium iodine (PI) to the culture medium 20 min before starting the experiment. Timelapse experiments measuring LIVE/DEAD fluorescence and thermal activation were performed in three sequential steps: (1) Hoechst channel (live cell label): 405 nm single-photon excitation confocal microscopy with a 415–567 nm emission window plus a transmitted brightfield channel; (2) PI channel (dead cell label): 488 nm single-photon excitation confocal microscopy with a 577–690 nm emission window; (3) pulsed IR multiphoton excitation at 750 nm attenuated to 1%, 6% or 10% of maximum power that corresponds to 5.5, 33 and 55 mW. Excitation was limited to a specific region of interest (ROI) where maximum attenuation of the IR laser was applied when scanning outside the chosen ROI area. NP multiphoton fluorescence was simultaneously measured with a 492–570 nm emission window. 4D Time-lapse experiments were performed applying the sequential acquisition described to capture Z-series composed of 12 or 14 sections at ~1.8 µm intervals captured approximately every 45 s.

### 2.5. In Vivo Experiments

Experiments were performed according to the ethical guidelines of our local ethical committee and consistent with national regulations for the care and use of laboratory animals (R.D. 53/2013).

#### 2.5.1. Teratogenicity Assay

This experiment was conducted following a protocol previously published by some of us [61]. On day 0, wild-type zebrafish were outcrossed. Later, embryos were collected and incubated for 4 h at 28 °C in E3 medium. Then, not-fertilized eggs were removed. At this point, healthy embryos were incubated with Fe@Au NPs suspended in E3 medium at different concentrations. A total of 20–30 eggs were placed in 8-well square Petri-dishes, with a final volume of 4 mL. Survival, hatching, and malformations were evaluated at different time points (24, 48, and 96 h) after exposure to NPs.

#### 2.5.2. In Vivo Pharmacokinetics and Biodistribution Experiments

Male Wistar rats (*n* = 3, ca. 220 g weight), provided by Janvier Labs, were used for all in vivo imaging experiments. Animals were anesthetized with isoflurane 1%. Two different experiments were conducted: (1) subdermal administration; (2) biodistribution and pharmacokinetics after intravenous administration. In the last case, the tail vein was cannulated, and the animals were placed into the imaging system (CT or MRI). Nanoparticles were administered in both cases at a concentration of 20 mg (Fe + Au) per kg.

#### 2.5.3. In Vivo Magnetic Resonance Imaging

MRI experiments were performed on the 9.4 T Bruker Biospec system described above, using a transmit-receive volume coil with an inner diameter of 72 mm. The acquisition and image analysis were performed following the protocol previously published by some of us [62], which is briefly described in the Supporting Information.

#### 2.5.4. In Vivo Computed Tomography (CT)

CT images were acquired on a Bruker Albira small animal CT system. Experimental details are similar to those described in CT in vitro. Pharmacokinetics were obtained by calculating the average HUs values within different ROIs located in the liver.

#### 2.5.5. Tumor Implantation

Six week old male nude mice (*n* = 3, ca. 22 g weight), provided by Charles River Labs, were used. C6 cells were brought to 80–90% confluence in culture. At this point, cells were trypsinized and pelleted at 4 °C. Cells were injected subcutaneously (1 × 10^5^ cells) in the mouse right flank.

#### 2.5.6. In Vivo Magnetic Resonance Imaging (MRI) of Tumor-Bearing Mice

The 40 mm quadrature bird-cage resonator was used for these experiments. When the tumor reached at least 0.125 cm^3^, NPs were administered intratumorally at a concentration similar to the one described above. The following acquisition scheme was used: T_2_-weighted, quantitative T_2_, subdermal injection of the magnetic NPs, T_2_-weighted, and quantitative T_2_. High-resolution T_2_-weighted images were acquired with a turbo-RARE sequence using the following parameters: (TE = 16 ms, TR = 1000 ms, 4 averages, 156 µm in-plane resolution, and 1 mm slice thickness) and also with respiratory gating. Quantitative T_2_ measurements were performed using a multi-echo spin-echo sequence, using the following parameters: (TEs ranging from 7 to 448 ms, TR = 3500 ms, FOV = 4 cm, matrix size = 128 × 128, slice thickness = 1 mm).

#### 2.5.7. Two-Photon In Vivo Photodynamic Therapy

Tumor irradiation was performed using a Leica SP5 MP inverted confocal microscope equipped with a Spectraphysics MaiTai HP IR laser for multiphoton excitation using a 10× HCX PL APO CS 0.40 NA objective lens. Pulsed IR multiphoton excitation at 720 nm was applied at maximum power (1.75 W laser output). Therapy was applied across a grid of 40 (8 × 5) 1600 µm^2^ scan fields using the LAS AF software Tilescanfunction. Each field was treated with 38 scans focused in 20 µm intervals toward progressively deeper tissue. This resulted in a final 11.3 × 7.17 × 0.76 mm^3^ treatment volume. Irradiation time was 1.725 min per 1600 µm^2^ field for a total irradiation time of 69 min.

#### 2.5.8. Histology

Histology was evaluated after tumor irradiation. Animals were sacrificed, and tumors were extracted. Histology samples were evaluated by light microscopy. Haematoxylin and Eosin (H&E) staining was used to assess tissue architecture. Detailed procedures are described in the Supporting Information.

### 2.6. Statistical Analysis

SPSS package (SPSS Inc., Chicago, IL, USA) was used for statistical analysis. The results are expressed as the mean ± standard deviation (SD). In order to determine statistically significant differences, Student’s *t*-test or one-way analysis of variance (ANOVA) were applied. The level of significance was *p <* 0.05.

## 3. Results and Discussion

### 3.1. Physicochemical Characterization of the NPs

**Figure 1:**
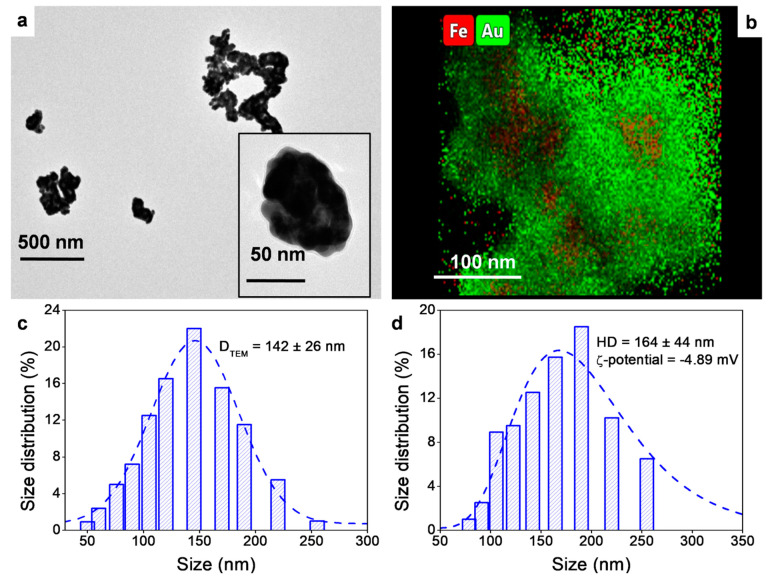
(**a**) Transmission electron microscopy images of the synthesized Fe@Au NPs. The inset is a 10× magnification of an isolated particle. (**b**) Spatial distribution of metal atoms in the NPs as measured by HAADF and EDX. (**c**) Size distribution obtained from the counting of 100 NPs in the TEM images. Gaussian fit is shown as a blue dashed line. (**d**) Size distribution and ζ-potential of the NPs suspension measured by DLS. Gaussian fit is shown as a blue dashed line.

Particle size, distribution, and elemental composition were evaluated by TEM and DLS. TEM data (Figure 1a) showed that Fe@Au NPs exhibit a spheroid shape with soft-spiky surface morphology, similar to a turtle shell structure. EDX of isolated particles confirmed the presence of both gold and iron (Figure S1). The spatial distribution of metal atoms was further measured by HAADF and EDX in STEM mode (Figure 1b), which confirmed a core-shell structure with a core entirely composed of Fe, whereas Au was mostly allocated at the periphery of the Fe nucleus. Further, STEM-EDX scan through a selected line showed a Fe peak at the center of the NP, while the Au trace had two side peaks (Figure S2).

TEM analysis of the particle size distribution indicated an average size of 142 ± 26 nm (Figure 1c). Additionally, the PVP capping stabilized the NP suspension in aqueous media. Their hydrodynamic diameter measured by DLS was 164 ± 44 nm (Figure 1d). Noteworthy, this size falls within the 10–200 nm range, which has been reported as the optimum for the bioaccumulation of NPs via enhanced permeability and retention (EPR) effect [31,63]. Size distributions agree with those reported in Quaresma et al. [54], and the slightly negative ζ-potential value of −4.89 mV indicates stability according to the DLVO theory [64]. The in vivo biodistribution of nanoparticles is highly dependent on the NPs surface characteristics, which in turn depend on the capping agent used to functionalize them. Therefore, a comprehensive evaluation of the PVP capping was performed. The FTIR spectrum of pure PVP showed characteristic bands, such as the stretching of the pyrrolidone C=O group (≈ 1650 cm^−1^) and the stretching C–N group (≈ 1290 cm^−1^) (Figure S3a). These peaks exhibited a shifting to lower wavenumbers, entailing some coordinative interactions between PVP and the NPs surface, and therefore confirming the PVP capping of the Fe@Au NPs, in agreement with previously reported FTIR analysis for PVP capping NPs [65]. Moreover, the TGA analysis revealed that the PVP capping represented only ≈ 2.5% of the total NPs mass (Figure S3b).

**Figure 2:**
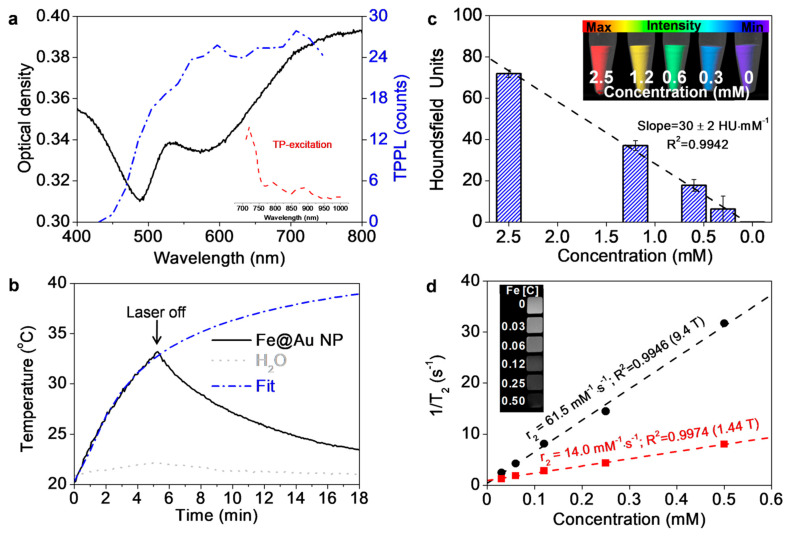
(**a**) Extinction spectra (dark line) and TPPL properties of the NPs suspension. Blue dashed line corresponds to TP-emission, while the inset represents the TP-excitation. (**b**) Phototermal conversion of the NPs. Black continuous line represents the temperature rise after laser radiation (1064 nm, 1.22 W/cm^2^) and heat release after laser switch-off. The temperature rise of a control water sample is plotted as a continuous grey line. Blue line is the fit of the experimental data to the analytical solution of the Fourier equation of a continuous point source. (**c**) X-ray attenuation intensity measurement calculated by the slope of the linear fit of CT values against NP concentrations. Inset: CT image of the different concentrations of Fe@AuNPs. (**d**) Plot of 1/T_2_ over nanoparticle concentration at 1.44 and 9.4 T. Inset: T_2_ weighted image of different NPs concentrations at 9.4 T. r_2_ values are drawn on each linear fit (dashed lines).

As for the optical properties of the NPs, the extinction spectrum of the Fe@Au in aqueous solution showed a strong absorption band peaked in the 700–800 nm range (Figure 2). This band is due to the gold LSPR and appears strongly broadened due to the multiple gold tips in the heterogeneous NP surface. This NIR long-tail wavelength range covers the NIR-I biological window and, therefore, ensures deeper penetration of radiation energy. This feature makes these NPs exceptionally suitable for biological studies [66,67].

After single-photon absorption, the de-excitation through nonradiative processes leads to local heating in the surrounding media. To evaluate the NIR photothermal conversion of these NPs, the extent of the energy released was assessed via optical hyperthermia experiments in aqueous NP dispersions at 1064 nm (see Figure 2b). The results demonstrated a quick temperature increase upon laser heating that stops after the laser is switched off. The contribution of the temperature increment due to water absorption was estimated to be ~1.3 K (dashed grey line in Figure 2b). The associated temperature increase rate was obtained by the linear fit of the experimental data within the first 2 min, while heating efficiencies were calculated with the temperature value at 5 min and the laser power. Water heating efficiency was only 1.5%, but in the presence of the NPs it increased to 14.2%. Similarly, the temperature increase rate of 3.3 °C∙min^−1^ was 10-fold higher for the NP suspension. Furthermore, the photothermal stability of Fe@Au NPs was evaluated by measuring the temperature while on/off of NIR irradiation for four cycles (5 min on followed by 20 min off) (Figure S4). These experiments demonstrated excellent stability of Fe@Au NPs under NIR irradiation since the maximum temperature level remained similar in all cycles. In addition, the calculated value for photothermal conversion efficiency was 42.6%. This value is superior or comparable to that obtained for other photothermal NP suspensions based on gold or CuS or organic-based nanosystems [58,68] and close to other magnetic NP suspensions [69]. Moreover, the temperature profile in one cycle was fitted to the analytical solution of the Fourier equation to a continuous point source in spherical coordinates. The fit corresponds to the dotted-dashed-line in Figure 2b and has a limiting steady-state temperature of T_∞_ = 46.5 K (∆T_∞_ ≈ 26 K). This temperature is in good agreement with the difference between the terminal (at a position *r*→∞) and the surface temperature calculated by solving the boundary problem with the finite element methods. The temperature mapping around the NP and the temperature along a radial segment from the NP center are shown in Figure S5. From the temperature increase rate at a time *t* = 2 min, the o-SAR is 12 kW∙g^−1^. This result is in good accord with other nanoplatforms reported recently in the literature [70].

TPPL properties of Fe@Au NPs were also measured as the first step towards their use in guided imaging therapy. As shown in Figure 2a, the NPs dispersion exhibited a TP-excitation spectrum with high levels of excitation between 710 and 750 nm, with a maximum at 720 nm, followed by a progressive decrease at longer wavelengths (see the inset in Figure 2a). After the identification of the optimal excitation condition (720 nm), we evaluated the emission spectrum. We found that Fe@Au NPs showed an increasing emission from 400 nm onwards, with a broad profile, as shown in Figure 2a. This wavelength window allowed us to set the detection range to 492–570 nm for TPPL microscopy and guided imaging therapy described below.

Finally, due to the composition of the as-prepared NPs, they present promising properties as multimodal CAs for both CT and MRI. Therefore, the X-ray attenuation coefficient of the Fe@Au NPs was measured at different NPs concentrations. As can be observed in Figure 2c, the plot of the attenuation coefficient in HU units against NPs concentration showed a slope value of 30 HU∙mM^−1^, which is more than 10-fold higher than the CAs commonly used in clinical practice, iohexol (2.7 HU∙mM^−1^) [71]. Moreover, transverse relaxivities at low (1.44 T) and high (9.4 T) magnetic fields were also measured. Fe@Au NPs exhibited r_2_ values of 14.0 and 61.5 mM^−1^∙s^−1^ at low and high magnetic fields, respectively (Figure 2d). Thus, Fe@Au NPs behave as efficient dual-modal imaging CAs. Indeed, their capabilities as CT and MRI CA, determined in vitro, improved those reported for other multimodal CAs by up to 200% [72,73].

### 3.2. In Vitro Cytotoxicity Assessment in Cell Cultures

Cellular biocompatibility is a key aspect for any nanoplatform intended for in vivo applications. Thus, a comprehensive evaluation of the cytotoxicity was accomplished considering different parameters such as cell morphology, mitochondrial activity, and the number of necrotic/late apoptotic cells. It is worth mentioning that to provide an appropriate assessment of the therapeutic effect and the potential side effects of Fe@Au NPs, both healthy and cancer cell models were used in these studies. Thus, human foreskin fibroblasts (HFF-1) and C6 rat glioma cells were selected as working models and exposed to increasing concentration of NPs in the 0.1–100 µg∙mL^−1^ range (Figures S6 and S7). Fe@Au NPs did not produce morphological changes in either of the cell models (Figures S4a–c and S5a–c), nor was a statistically significant decrease in the total number of cells observed for any of the concentrations studied (*p* < 0.05) (Figures S6d and S7d). No significant increase in the percentage of dead cells was observed, either (*p* < 0.05) (Figures S6e and S7e). As for the mitochondrial activity, the MTT assays showed that it remained above 70% for all concentrations studied, which is a reasonable value for this type of assay according to ISO norms (ISO-10993-5) (Figures S6f and S7f).

### 3.3. In Vitro Photoluminescence and Photothermal Therapy

**Figure 3:**
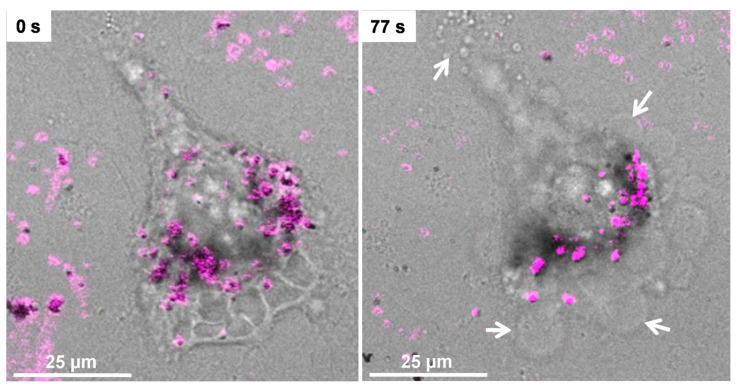
Representative field of C6 cells incubated for 24 h with 100 µg∙mL^−1^ of Fe@Au NPs and irradiated with 750 nm IR laser (6% power), showing the merge of brightfield and 2P photoluminescence emissions before (left) and after (right) irradiation. Arrows indicate loss of plasma membrane integrity.

The TPPL properties of Fe@Au NPs demonstrated that they fit the requirement for promising cell labeling probes and potential use for cancer therapy by light-to-heat conversion in the NIR region. In particular, the maximum TP-excitation wavelength relies on the broad single-photon absorption region of the NPs, a fact that enables a subtle energy tuning to both imaging at low irradiation power and PTT at higher fluences via photothermal conversion. Therefore, we assessed these features in 2D and spheroidal 3D regions of C6 cultures. The latter provides a valuable approach that bridges the gap between traditional cell cultures and tissues [74]. In the 2D arrangement, no appreciable effect was observed on cell morphology, even after long-term exposure to laser radiation of 750 nm at 1% laser output, while TPPL was readily detectable (Figure S8). However, by increasing the laser output to 6%, we found that Fe@Au NPs induced necrotic features such as an increment of the cell volume, loss of plasma membrane integrity, and leakage of cellular content, after short-term irradiation (Figure 3 and Video S1).

**Figure 4:**
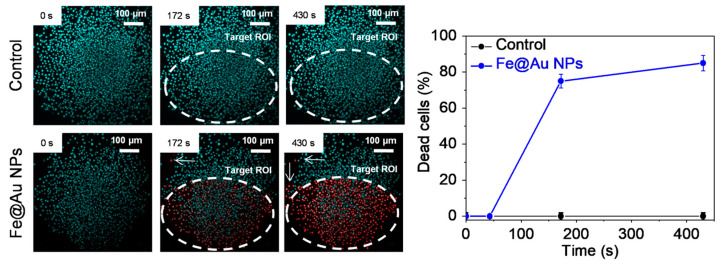
Left: representative images of C6 cells at different irradiation times. The TPPL process was triggered with 720 nm laser output at 10%. Top images correspond to cells exposed for 24 h to PBS and bottom images represent cells exposed for 24 h to Fe@Au NPs. The images show the merge of DAPI (blue) and PI (red) channels. Right: quantitative evaluation of dead cells percentage induction by laser irradiation.

Similar features were found in the 3D model, as sketched in Figure 4 (Video S2). Multiple photon irradiation was limited to a target ROI, while single-photon detection of the LIVE/DEAD staining of DNA (blue; Hoechst) and dead cells (red; PI) provided a real-time readout of cell mortality in the whole tissue region. After 172 s of irradiation, ~75% of the cells in the target ROI were dead, while a low rate of cell death was observed outside this area. These findings are indicative of local heating and photo-induced radical formation that may damage the closest neighboring cells. After 430 s, the percentage of dead cells reached 90% inside the ROI and remained low (<1%) in the peripheral region. It ought to be mentioned that, even for longer irradiation times, NPs-free cultures presented no traces of light-induced increment of cell death (Figure S9).

### 3.4. In Vivo Toxicity Evaluation on Zebrafish Embryos

**Figure 5:**
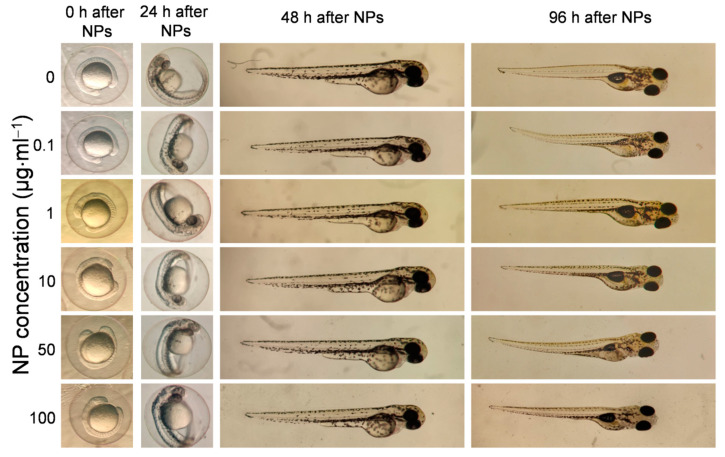
Morphological analysis of zebrafish embryos exposed to different concentrations of Fe@Au NPs at different times (0, 24, 48, and 96 h).

The zebrafish model has been proposed as an appropriate intermediate screening step to properly evaluate the toxicity of a given compound [61]. This approach relies on the evaluation of the hatching, survival rates, and body malformations of zebrafish embryos exposed to different concentrations of Fe@Au NPs (0.1, 1, 10, 50, and 100 μg∙mL^−1^) at different times post-exposure. At 24 h after exposure, control embryos exhibited around 30% of hatching, which can be described as normal hatching values for zebrafish embryos [75]. At this time point, a dose-dependent trend was observed. Higher concentrations of Fe@Au NPs produced an increase in hatching rate of up to 50%, compared to control nonexposed embryos (Figure S8a). This early hatching may be explained by the adsorption of NPs on the chorion, which blocks the pores with the consequent restriction of oxygen and nutrients [76]. In fact, Fe@Au NPs were clearly observed interacting with the chorion of the embryos (Figure S10b). Despite the observed effect over hatching, neither malformations nor mortality were observed over time in embryos exposed to different doses of Fe@Au NPs (Figure 5 and Figure S11).

### 3.5. Pharmacokinetics and Biodistribution Analysis

**Figure 6:**
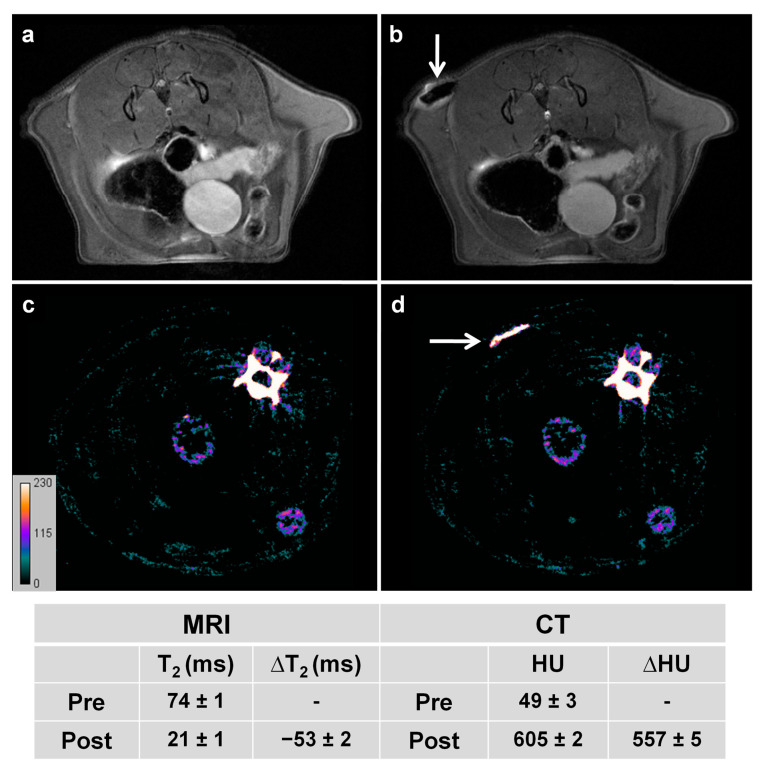
Representative in vivo images before (**a**,**c**) and after (**b**,**d**) subcutaneous administration of Fe@Au NPs. Top: T_2_-weighted images; bottom: CT images. Arrows indicate NPs location. The table collects the corresponding ΔT_2_ and ΔHU values.

Fe@Au NPs demonstrated in vitro biocompatibility and excellent features as CAs for MRI and CT techniques. The next step taken to evaluate the in vivo CA efficiency of the NPs was to verify the presence of NPs at a therapeutic dose inside the tumor, which is crucial for successful therapy. Local administration (or imaging-guided therapy approach) was first evaluated by MRI and CT (Figure 6). After subcutaneous administration, MRI studies revealed a strong darkening within the injected zone (Figure 6a,b). Similarly, the CT studies showed a substantial enhancement of X-ray attenuation (Figure 6c,d). The quantitative analysis demonstrated substantial variations of contrast, with ∆T_2_ of −52 ms and ∆HU of 557 HU, which confirmed the multimodal CA properties of Fe@Au NPs after local administration.

**Figure 7:**
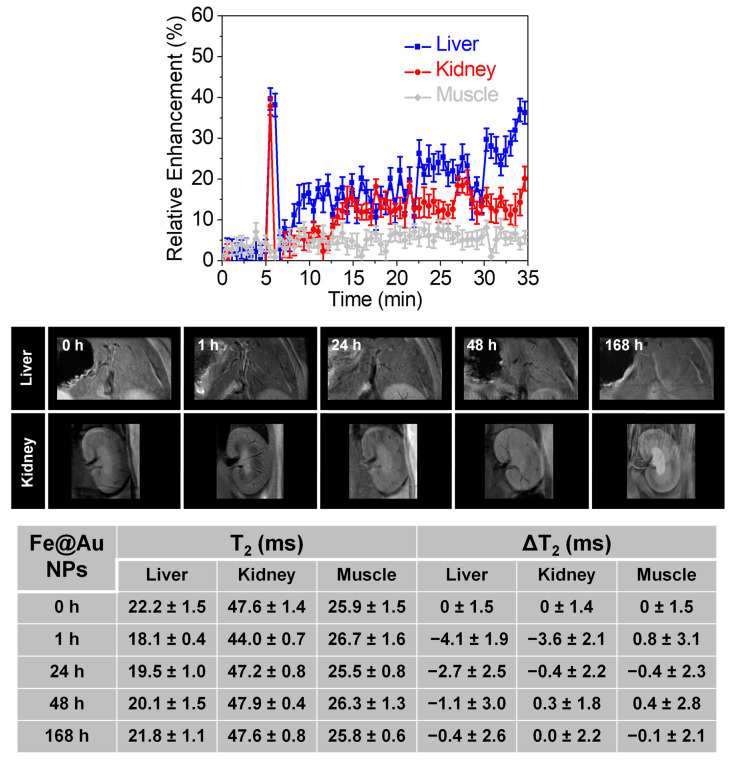
(**top**) In vivo pharmacokinetics after intravenous injection in mice of Fe@Au NPs in the liver (blue), kidneys (red), and muscle (grey). (**bottom**) Representative T_2_-weighted MRI images of liver and kidneys at different time points after the intravenous injection of NPs. The table shows T_2_ and ΔT_2_ values in the liver, kidneys, and muscle at different time points after the intravenous injection of Fe@Au NPs. Values correspond to the mean ± SD (*n* = 3).

On the other hand, both short and long-term pharmacokinetics [77], and the therapeutic effect, of Fe@Au NPs after intravenous administration were evaluated by MRI and CT. Dynamic MRI was used to assess the short-term behavior of Fe@Au NPs over the first 35 min in the liver, kidneys, and muscle (used as control tissue due to its typically low NPs uptake) [78]. Relative enhancement (RE) curves for liver and kidneys exhibited a similar trend, with an abrupt increment of around 40% after the injection, followed by a fast decrease to the basal state. Then, both RE curves showed a slow increase in the signal to 40% and 20% for liver and kidneys, respectively (Figure 7 top).

No significant variation in the RE curve was reported for the muscle, as expected. Thus, NPs crossed through the liver and kidneys in two steps: a fast pass without retention, followed by a slow uptake that is more effective in the liver. It is worth noting that the uptake profile of the Fe@Au NPs reported here is much slower than that of other magnetic nanoparticles previously reported by us with similar and even smaller sizes [78]. This behavior suggests that PVP bestows better stealth properties than PEG, the capping agent used in our previous studies. Long-term pharmacokinetics were evaluated by qualitative T_2_-weighted images and the corresponding quantitative T_2_ mapping at 0, 1, 24, 48, and 168 h (Figure 7 bottom). Liver and kidneys showed strong darkening 1 h post-administration of Fe@Au NPs that corresponded to a T_2_ variation (ΔT_2_) of −4.1 and −3.6 ms for liver and kidney, respectively. The brightness was partly recovered at 24 h in the liver, with ΔT_2_ of −2.7, and completely retrieved to the basal state at 48 h post-injection. As for the kidneys, the basal state was reached at 24 h. Previous reports showed that minimal retention occurs in the kidneys for NPs sizes above 100 nm HD [79]. Therefore, the T_2_ decay observed in the kidneys was very likely due to circulating NPs, implying that Fe@Au NPs were completely cleared from the bloodstream after 24 h. This hypothesis was confirmed by the ICP-MS analysis performed at 24 h post-injection, which demonstrated the absence of NPs in the blood and kidneys, being only detectable in the liver and spleen (Figure S12). Similar results were obtained in the CT experiments. As observed in Figure S13, a considerable increase in the contrast was observed 1 h post-administration. This increase was almost completely attenuated at 24 h, with a recovery to baseline at 48 h post-injection. Quantitative analysis revealed ΔHU of 28.1 at 1 h, with a decrease to ΔHU 7.8 at 24 h post-administration. It is worth mentioning that the long-term pharmacokinetics was followed up here for NPs concentrations 20-times smaller than the recommended average clinical dose for iohexol (https://www.drugs.com/dosage/iohexol.html#Usual_Adult_Dose_for_Body_Imaging, accessed on September 2020). Finally, potential side effects associated with systemic toxicity after intravenous administration of Fe@Au NPs were checked by analysis of the weight profile and ex vivo histological evaluation of the main organs at 168 h post-administration. The weight profile showed a decrease in body weight at 24 h post-administration of NPs, which was also noticeable in the control group. This effect could be related to anesthesia and manipulation processes. Throughout the experiments, both control and injected groups experienced similar behavior, which suggests the absence of toxicity in vivo (Figure S14). Histological assessment was performed on H&E stained sections of the liver, kidneys, spleen, and heart to evaluate the organ architecture. Liver sections did not exhibit any appreciable change compared with control tissue, i.e., normal hepatocytes and Kupffer cells were observed without evidence of necrosis or apoptosis. Similarly, no substantial changes were observed in the comparative observation of the rest of the main organs (Figure S15).

Overall, the results reported here demonstrate that Fe@Au NPs undergo an uptake-metabolism-excretion process within the liver of about 48 h and have blood circulation times below 24 h. Therefore, since the optimum accumulation via EPR effect is expected to occur between 24 and 48 h after injection [80], the accumulation of Fe@Au NPs in a therapeutic dose inside the tumor would be hampered by their short blood residence time, along with the avid liver uptake. Consequently, local administration was selected for in vivo TP-photothermal therapy and imaging-guided therapy experiments.

### 3.6. In Vivo Imaging-Guided TP-Photothermal Therapy

**Figure 8:**
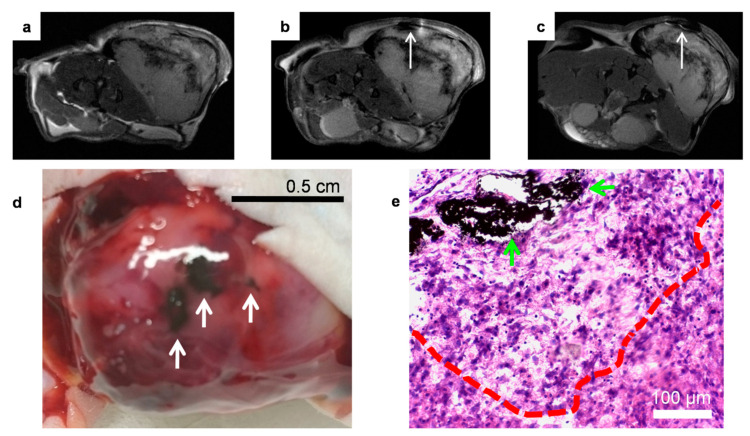
Representative in vivo T_2_-weighted images of a C6 tumor before (**a**) and after (**b**) intratumoral administration of Fe@Au NPs, and after irradiation (**c**). (**d**) Color photograph of the irradiated tumor. White arrows indicate dark areas related to NP location or NP-induced necrotic areas. (**e**) H&E stained histological sections of the tumor after irradiation. Green arrows indicate the location of the NPs and the red line delimits the area affected by the photothermal conversion process.

After discerning the best NPs administration route and taking advantage of their appealing TPPL properties, the in vivo imaging-guided therapeutic efficacy of Fe@Au NPs was evaluated. One of the main advantages of our confocal TP-microscope is the possibility of accurately selecting the irradiation depth, which results in more precise control, consequently limiting the potential side effects. The PTT efficiency was evaluated after irradiation at 720 nm. This wavelength corresponds to the maximum TP-excitation wavelength and is also in resonance with the broad LSPR band. An obvious darkening was observed by MRI after NPs administration (Figure 8a,b). In addition, after PTT treatment, evident changes around the location of the NPs injection area were observed as a notable increase of the dark region (Figure 8c). Black areas were visible even to the naked eye (Figure 8d). However, since Fe@Au NPs have a dark bluish color, histological analysis is required to differentiate between necrotic zones and NPs cumulus. Thus, histological evaluation of H&E stained tumor sections revealed that cells were missing in the neighboring area of the NPs allocation, with some holes being observable in the tissue (Figure 8e). Additionally, severe cellular damage, with large necrotic areas, was detected up to 300 µm away from NPs clusters. In these zones, deterioration of the nuclear membrane and shrinkage of nuclei, with pyknosis and/or karyorrhexis, was observed [81]. Our results are quantitatively similar to those reported for TP-PTT following intratumoral injection of silver NPs [82]. On the other hand, although remarkable results have been achieved in cancer theranostics with hyaluronic acid-modified Fe_3_O_4_@Au core-shell nanostars [83], they present some important limitations related to the lack of control of the irradiated volume and the power loss caused by absorption/scattering of tissues in the laser trajectory. Thus, in this work, we used a confocal microscope, which allowed us a more precise and 3D control of NIR irradiation, and therefore limiting the potential side effects, as the damage of healthy tissues, in the treatment of more infiltrating tumors.

While a successful imaging-guided PTT was confirmed, some limitations remain to be overcome to enable efficient tumor targeting via venous route. Among them, size reduction and active targeting are the main challenges to be solved by surface engineering and alternative synthetic routes of the NPs, which are currently underway.

## 4. Conclusions

Cytocompatible iron oxide-gold NPs with core-shell structure were synthesized by a seed-mediated growth chemical method. Core-shell arrangement was confirmed by TEM and HAADF-EDX in STEM mode. The PVP capping bestowed the Fe@Au NPs with water dispersibility and stealth properties, making them suitable for biomedical purposes. In addition, these NPs reported attractive r_2_ relaxivity and X-ray attenuation properties. Both cytotoxicity assays in cell cultures and in vivo toxicity studies in zebrafish embryos showed very low toxicity of the synthesized Fe@Au NPs.

In rodent models, optimization of the administration route showed that although intravenous administration exhibited negligible side effects, pharmacokinetics and biodistribution studies demonstrated that this route strongly limited the possibility of accumulating a therapeutic dose inside the tumor. Therefore, the intratumoral approach was selected for imaging-guided photothermal therapy. The roughness of the gold shell, its broad and strong LSPRs in the NIR absorption, and photoconversion rate enabled their use as PTT agents. In particular, TPPL features were exploited in an in vivo proof of concept of imaging-guided PTT after intratumoral administration. This experiment constitutes one of the few attempts to explore the TPPL properties of NPs for in vivo PPT. After the focused TP-NIR irradiation process, evidence of local tumor tissue damage was revealed by MRI and confirmed by histology. Therefore, this work presents a starting point for the near-future development of new multimodal nanoplatforms for cancer theranostics.

## Data Availability

The data presented in this study are available on request from the Corresponding author.

## Supplementary Materials

The following are available online at https://www.mdpi.com/1999-4923/13/3/416/s1, Figure S1: Representative EDX spectrum of Fe@Au NPs, Figure S2: EDS elemental mappings of Fe@Au NPs, Figure S3: FTIR analysis of PVP (blue) and Fe@Au NPs (red), Figure S4: Photothermal stability of Fe@Au NPs, Figure S5: Temperature map around a stratified NP structure with geometry and composition extracted from the experimental data (top), Figure S6: Representative images of HFF-1 cells, Figure S7: Representative images of C6 cells, Figure S8: Representative field of C6 cells incubated for 24h with 100 µg⋅mL^−1^ of Fe@Au NPs and irradiated with 750 nm IR laser, Figure S9: Representative images of C6 cells exposed for 24 h to PBS after irradiation with a 720 nm laser fixed at 10% power, Figure S10: Hatching rate of zebrafish embryos exposed to Fe@Au NPs for 24 h, Figure S11: Survival rate of zebrafish embryos exposed to TbNRs at 48 h post-exposure, Figure S12: ICP–MS quantification of Au content at 24h post intravenous injection of Fe@Au NPs, Figure S13: Representative in vivo CT images at different experimental times (0, 1, 24, 48, and 168 h) after the intravenous injection of Fe@Au NPs, Figure S14: Relative weight profile of mice intravenously injected with PBS (black) and Fe@Au NPs (grey), Figure S15: H&E staining of representative histological sections of liver, kidneys, spleen, and heart, Methods content.
