# Supplementary material for: Fe3O4-Au Core-Shell Nanoparticles as a Multimodal Platform for In Vivo Imaging and Focused Photothermal Therapy
Source: Pharmaceutics. 2021 Mar 20;13(3):416. doi: 10.3390/pharmaceutics13030416 (PMC8003746; doi:10.3390/pharmaceutics13030416)
Supplement: Supplementary file 1 [file pharmaceutics-13-00416-s001.pdf]

pharmaceutics

*Supplementary Materials*

# $Fe_3O_4$-Au Core–Shell Nanoparticles as a Multimodal Platform for in vivo Imaging and Focused Photothermal Therapy

**Carlos Caro, Francisco Gámez, Pedro Quaresma, Jose María Páez-Muñoz, Alejandro Domínguez, John R. Pearson, Manuel Pernía Leal, Ana M. Beltrán, Yilian Fernandez-Afonso, Jesús M. De la Fuente, Ricardo Franco, Eulália Pereira and Maria Luisa García-Martín**

**Index**

## 1. Methods

### *1.1. Synthesis of the $Fe_3O_4$@Au Core–Shell NPs*

In the first step, magnetic NPs were solubilized by citric acid functionalization according to the (slightly modified) protocol of Lattuada and Hatton [1]. Briefly, 0.3 g of citric acid was added to a round-bottom flask with 36 mg of magnetic NPs suspended in 22.5 mL of DMF. The mixture was heated to 100 °C for 12 h under vigorous stirring, followed by the addition of about 20 mL of ethyl ether. The particles were removed from the reaction mixture by magnetic separation. The solid was further washed using 3 acetone redispersion/magnetic separation cycles. Finally, the powder was dried in vacuum for 2 h at room temperature.

In a second step, gold seeds were deposited on the solubilized magnetic NPs. In a round-bottom flask, 680 µL of a 35 mM $HAuCl_4$ aqueous solution was added to 100.5 mL of 5.98 mM $NH_3$ aqueous solution that contained 5 mg of citrate treated NPs. The mixture was heated and kept at 40 °C for 15 min, followed by magnetic separation and redispersion of the NPs in 100 mL of water.

Reduction step with $NaBH_4$. 1 mL of a 0.1 M $NaBH_4$ solution was added to 100 mL of $Fe_3O_4$ NPs coated with gold seeds. After 1 min, NPs were magnetically separated and washed twice with water. Finally, the NPs were redispersed in a 10 mM solution of PVP in ethanol.

Growth of star-shaped gold shells. $HAuCl_4$ (866 µL of 20 mM aqueous solution) was added to 100 mL of a 10 mM PVP solution in DMF. After ~2 min (time needed to reduce the Au(III) to Au(I) [2], 6.6 mL of the previously prepared Au seed-coated magnetite NPs dispersed in ethanol were added. The mixture was sonicated with an ultrasound tip for 30 min. Magnetic particles were separated with a magnet and redispersed in 10 ml of water. The washing cycles were repeated in triplicate to remove excess PVP.

### *1.2. Cell Morphology Studies*

HFF-1 or C6 cells were plated at a density of $1 \times 10^4$ cells/well in a 96-well plate at 37 °C in 5% $CO_2$ atmosphere (100 µL per well). After 24 h of culture, the medium in the wells was replaced by fresh media containing the magnetic NPs in varying concentrations from 0.1 to 100 $\mu g \cdot mL^{-1}$ (Fe+Au). After 24 h, Triton X-100 was added to the positive control wells. All wells were stained after 15 min with: 1) DAPI (4′,6-diamidino-2-phenylindole, dilution 1:3000 in PBS) to label cell nuclei, although with stronger labeling in live cells; 2) calcein (1:1000 in PBS) to evaluate cell activity and 3) TO-PRO-3 iodine to label dead cells (dilution 1:1000 in PBS). Cell morphology images were acquired with a Perkin Elmer Operetta High Content Imaging System with a 20 × LWD 0.45 NA air objective lens. Five well replicates for each condition were analyzed with 10 random image fields captured per well. For each field, fluorescence images for DAPI, calcein-AM, and TO-PRO-3, plus a

**Publisher's Note:** MDPI stays neutral with regard to jurisdictional claims in published maps and institutional affiliations.

**Copyright:** © 2021 by the authors. Licensee MDPI, Basel, Switzerland. This article is an open access article distributed under the terms and conditions of the Creative Commons Attribution (CC BY) license (http://creativecommons.org/licenses/by/4.0/).

brightfield image, were captured. Cell mortality percentages were calculated automatically by Operetta Harmony software, whereby all nuclei (dead and alive) were identified from the DAPI staining and the percentage of dead cells then determined by the number of nuclei presenting high levels of TO-PRO-3 staining. Intracellular esterase activity was evaluated by calcein-AM staining.

### *1.3. Cytotoxicity Assays*

Briefly, the HFF-1 or C6 cells were plated at a density of $1 \times 10^4$ cells/well in a 96-well plate (37 °C, 5% $CO_2$, 200 µL per well, number of repetitions = 5). After 24 h of culture, the media in the wells was replaced with fresh media containing SPIONs in varying concentrations from 0.1 to 100 µg·mL$^{-1}$. At 24 h, the supernatant of each well was replaced by 100 µL of fresh medium with 3-[4,5-dimethylthiazol-2-yl]-2,5-diphenyl tetrazolium bromide (MTT) (0.5 mg·mL$^{-1}$). After 2 h of incubation at 37 °C and 5% $CO_2$ the medium was removed, and the formazan crystals were solubilized with 200 µL of DMSO, and the solution was vigorously mixed to dissolve the reacted dye. The absorbance of each well $[Abs]_{well}$ was read on a microplate reader (Dynatech MR7000 instruments) at 550 nm. The relative cell viability (%) and its error related to control wells containing cell culture medium without nanoparticles were calculated by the Equations (1) and (2):

$$RCV(\%) = \left(\frac{[Abs]_{test} - [Abs]_{Pos.Ctrl.}}{[Abs]_{Neg.Ctrl.} - [Abs]_{Pos.Ctrl.}}\right) \times 100 \quad (1)$$

$$Error(\%) = RCV_{test} \times \sqrt{\left(\frac{\sigma_{test}}{[Abs]_{test}}\right)^2 + \left(\frac{\sigma_{\mathrm{Ctrl}}}{[Abs]_{Ctrl.}}\right)^2} \quad (2)$$

where σ is the standard deviation. Triton X-100 was added to the positive control wells.

### *1.4. In vivo Magnetic Resonance Imaging*

High-resolution $T_2$-weighted images were acquired using a turbo-RARE sequence with respiratory gating (TE = 16 ms, repetition time TR = 1000 ms, 2 averages, 320 µm in-plane resolution, and 2 mm slice thickness). Quantitative $T_2$ measurements were also performed using a multi-echo spin-echo sequence (TEs ranging from 7 ms to 224 ms, TR = 3000 ms, FOV = 4 cm, matrix size = 160 × 128, slice thickness = 2 mm). The time-courses were followed by using a turbo-RARE sequence (TE = 16 ms, TR = 1000 ms, 1 average, 156 µm in-plane resolution, and 1 mm slice thickness) with a temporal resolution of 30 s. For subdermal administration, the acquisition scheme was as follows: $T_2$-weighted, quantitative $T_2$, subdermal injection of magnetic NPs, $T_2$-weighted, and quantitative $T_2$. For intravenous administration, the acquisition scheme was as follows: $T_2$-weighted, quantitative $T_2$, intravenous injection of the magnetic NPs, dynamic $T_2$-weighted sequence for 35 min, $T_2$-weighted and quantitative $T_2$. The dynamic image sequence was analyzed semi-quantitatively using the following equation:

$$RE = \left|\frac{I_t - I_0}{I_0} \times 100\right| \quad (3)$$

where RE is the modulus of relative signal enhancement, $I_t$ is the signal intensity at any given time after the NPs injection, and $I_0$ is the signal intensity before the injection.

Long-term pharmacokinetics were measured by quantitative $T_2$ mapping at 0, 1, 24, 48, and 168 h. Pharmacokinetics were obtained by calculating the average values within different ROIs in the liver, spleen, kidneys, and muscle.

### *1.5. In Vitro Transversal Relaxivities ($r_2$)*

Regions of interest (ROIs) were drawn on the first image of the image sequence, and the intensity values extracted and fit to the following equations:

$$M_Z(t) = M_0(1 - e^{-TR/T_1}) \quad (4)$$

$$M_{XY}(t) = M_0 e^{-TE/T_2} \quad (5)$$

where $M_Z$ and $M_{XY}$ are the signal intensities at time *TR* (repetition time) or *TE* (echo time), and $M_0$ is the signal intensity at equilibrium.

### *1.6. Histology*

The tissues were fixed in 4% formaldehyde (Panreac, pH 7 buffered) for 48 h, changing the 4% formaldehyde after 24 h. The samples were then dehydrated through graded ethanol and embedded in paraffin (temperature 56 °C for 2 h under stirring and vacuum). The detailed procedures are described below.

Hematoxylin and eosin (H&E): paraffin-embedded samples were sectioned at 7 µm thickness, then deparaffinized, rehydrated, and stained with H&E, and finally dehydrated in ascending concentrations of ethanol, cleared in xylene, and mounted on commercial glass slides.

## **2. Results**

### *2.1. Physicochemical Characterization of the Nanoparticles*

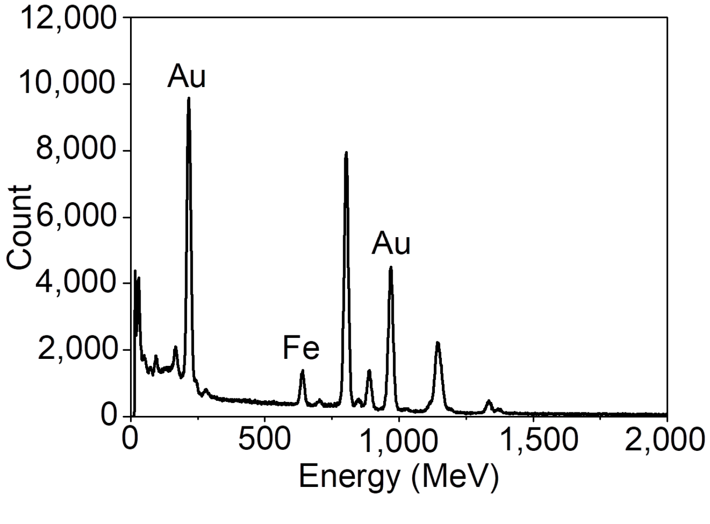


**Figure S1.** Representative EDX spectrum of Fe@Au NPs.

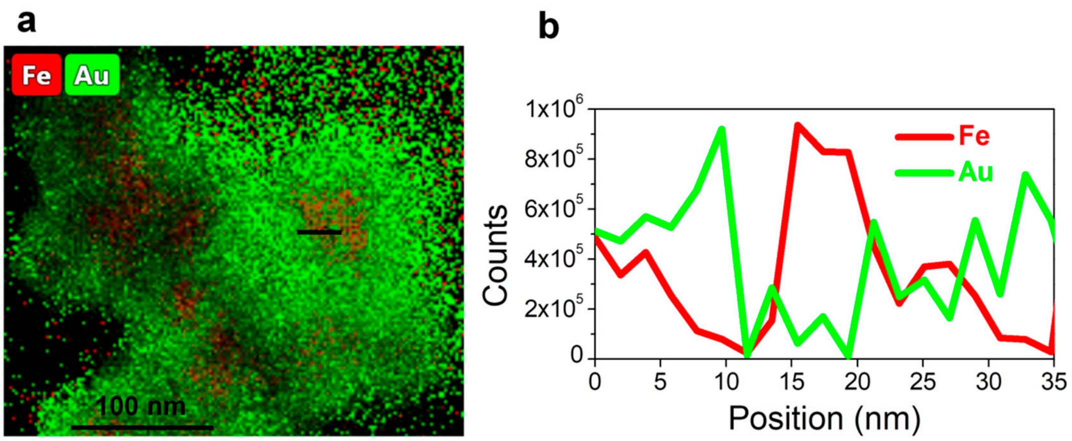


**Figure S2.** EDS elemental mappings of Fe@Au NPs. Green represents Au component, and red represents Fe component (**a**). Corresponding EDS line-scan profile (**b**). The scanned segment is indicated as a black arrow pointing the scanning direction in the left image.

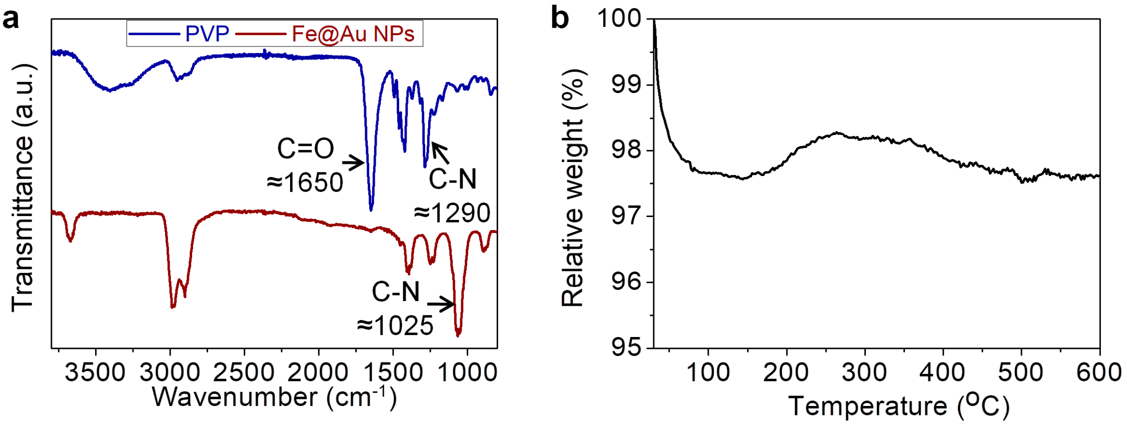


**Figure S3.** (**a**) FTIR analysis of PVP (blue) and Fe@Au NPs (red). (**b**) TGA spectrum of Fe@Au NPs.

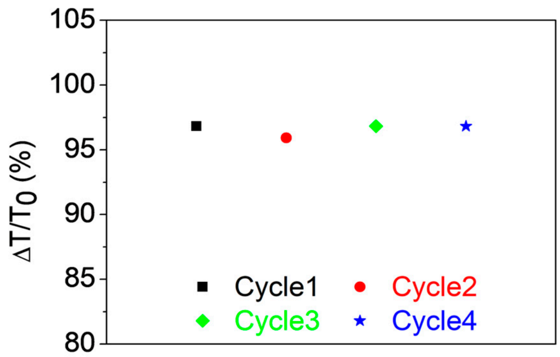


**Figure S4.** Photothermal stability of Fe@Au NPs.

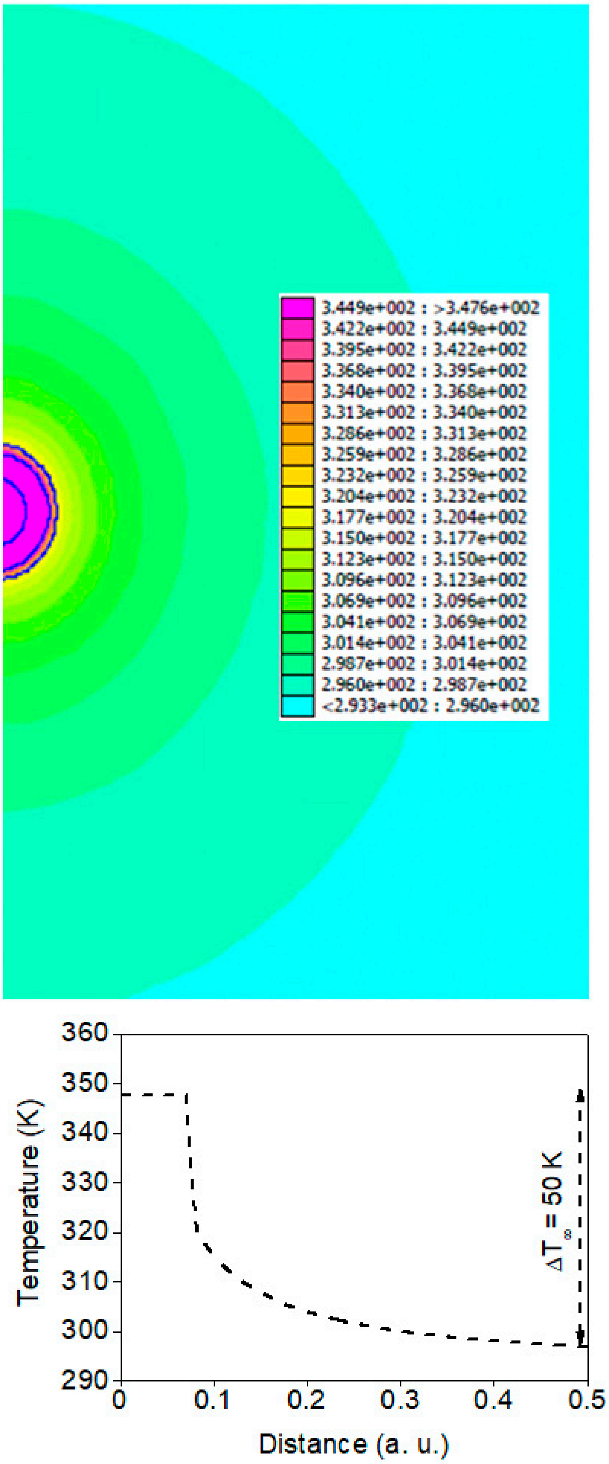


**Figure S5.** Temperature map around a stratified NP structure with geometry and composition extracted from the experimental data (top). The temperature profile through a radial coordinate is shown in the bottom figure. The surface-to-terminal temperature is calculated to be $\Delta T_\infty = 50$ K.

## 2.2. In Vitro Cytotoxicity Assessment in Cell Cultures

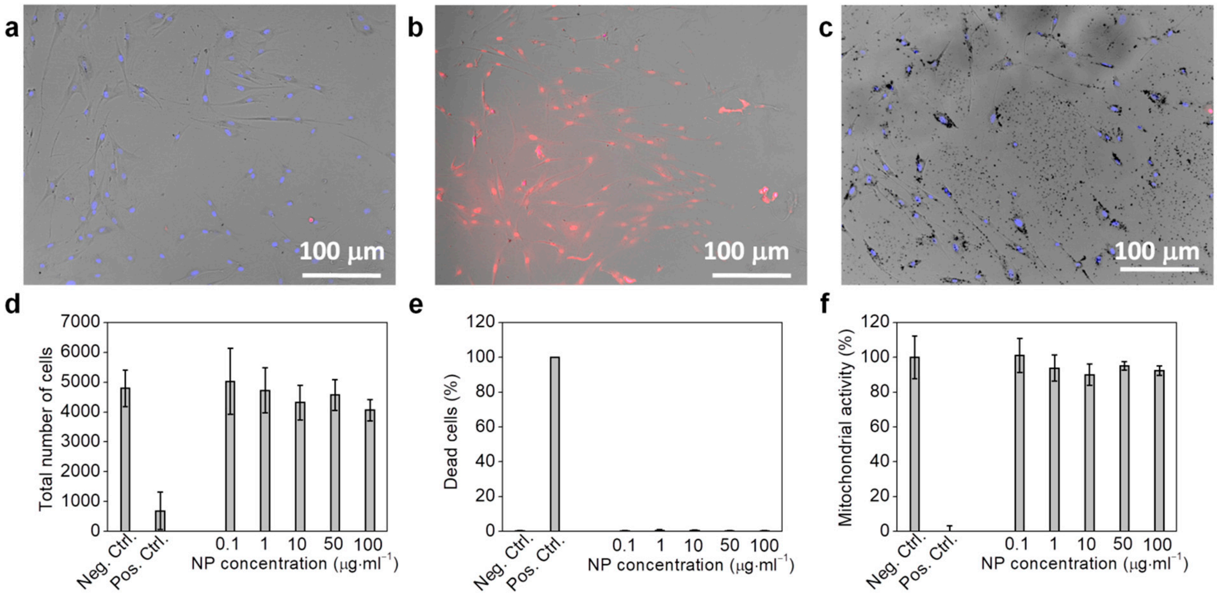


**Figure S6.** Representative images of HFF-1 cells: (**a**) negative control, (**b**) positive control, (**c**) cells exposed to 100 μg·mL$^{-1}$ of Fe@Au NPs. The images show the merged brightfield (grey), DAPI (blue), and TO-PRO-3 iodine (red) images. Scale bar is 100 μm. (**d**) Total number of cells per well exposed to increasing concentration of Fe@Au NPs. (**e**) Percentage of dead cells exposed to increasing concentration of Fe@Au NPs. (**f**) MTT assay of cells exposed to increasing concentration of Fe@Au NPs.

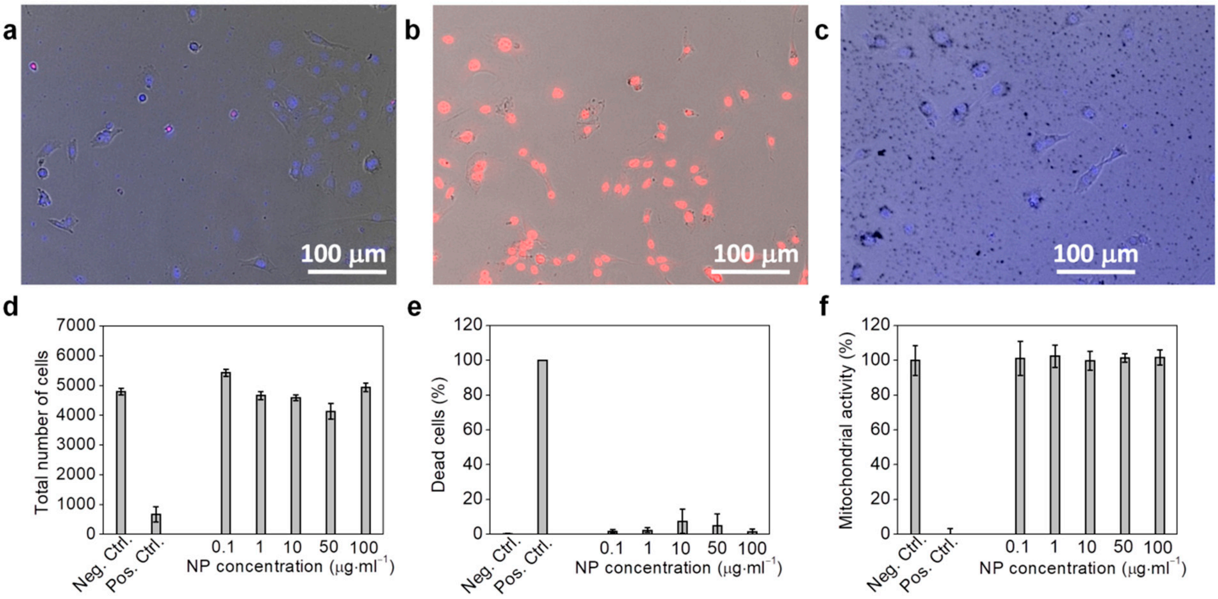


**Figure S7.** Representative images of C6 cells: (**a**) negative control, (**b**) positive control, (**c**) cells exposed to 100 μg·mL$^{-1}$ of Fe@Au NPs. The images show the merged brightfield (grey), DAPI (blue), and TO-PRO-3 iodine (red) images. Scale bar is 100 μm. (**d**) Total number of cells per well exposed to increasing concentration of Fe@Au NPs. (**e**) Percentage of dead cells exposed to increasing concentration of Fe@Au NPs. (**f**) MTT assay of cells exposed to increasing concentration of Fe@Au NPs.

## 2.3. *In Vitro Photoluminescence and Photothermal Therapy in Cell Cultures*

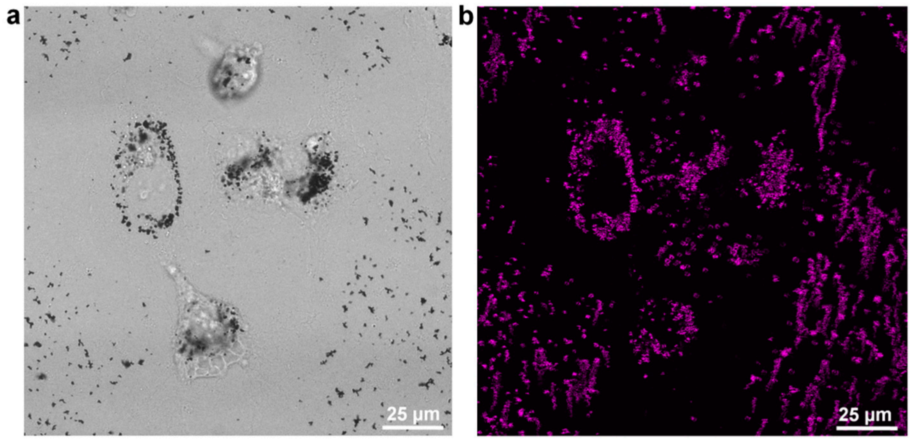


**Figure S8.** Representative field of C6 cells incubated for 24h with 100 µg·mL⁻¹ of Fe@Au NPs and irradiated with 750 nm IR laser. (**a**) brightfield and (**b**) TP fluorescence emission between 411-656 nm. See Materials and Methods for details.

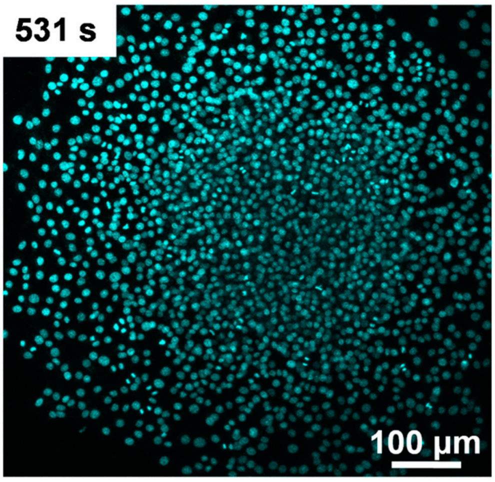


**Figure S9.** Representative images of C6 cells exposed for 24 h to PBS after irradiation with a 720 nm laser fixed at 10% power. The image shows the merge of DAPI (blue) and PI (red) channels.

## 2.4. *In Vivo Toxicity Evaluation on Zebrafish Embryos*

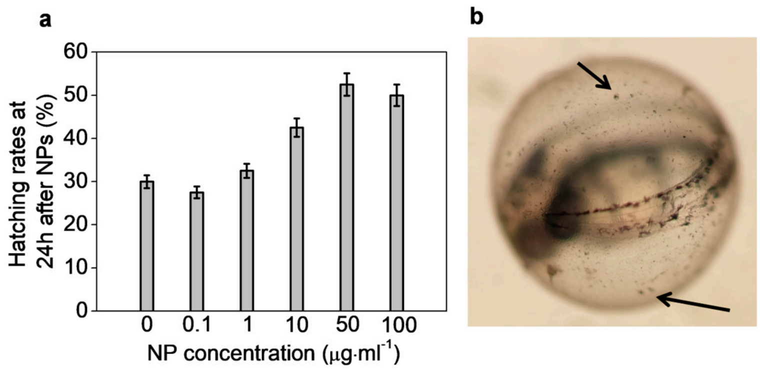


**Figure S10.** Hatching rate of zebrafish embryos exposed to Fe@Au NPs for 24 h (**a**). Hatching rates are expressed as the mean ± SD by analyzing 20–30 eggs per NP concentration. Representative images of zebrafish embryos exposed for 24 h to 100 µg·mL⁻¹ of Fe@Au NPs (**b**).

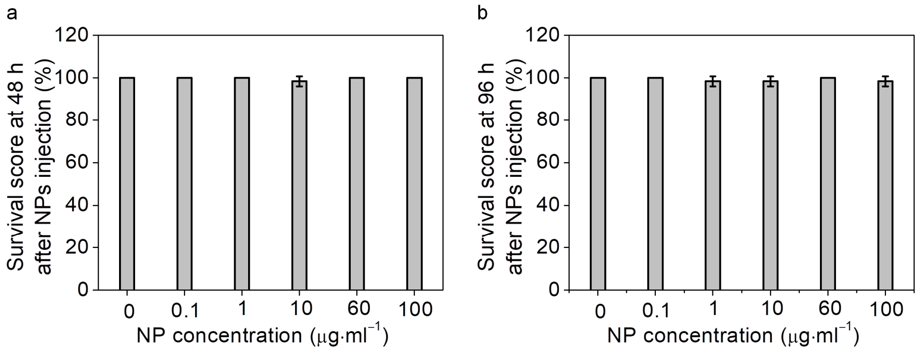


**Figure S11.** Survival rate of zebrafish embryos exposed to TbNRs at 48 h post-exposure (**a**) and at 96 h post-exposure (**b**). Data are expressed as the mean ± SD by analyzing 20–30 eggs per NP concentration.

## *2.4. In Vivo Pharmacokinetics and Biodistribution*

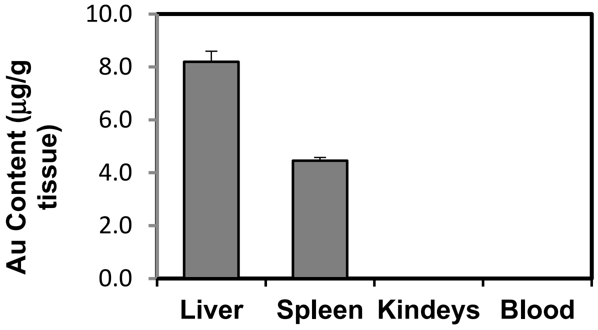


**Figure S12.** ICP–MS quantification of Au content at 24h post intravenous injection of Fe@Au NPs. The liver has been highlighted for better observation. Error bars correspond to the standard deviation.

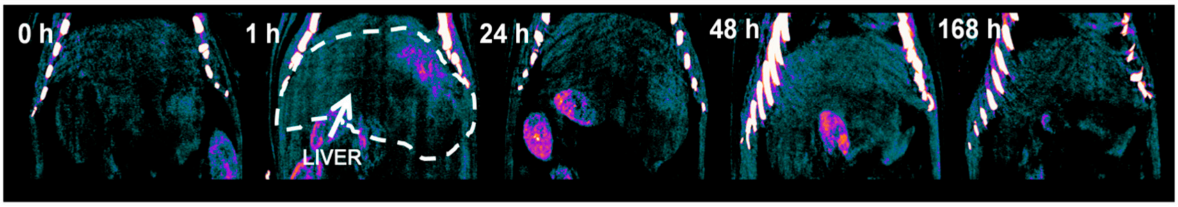


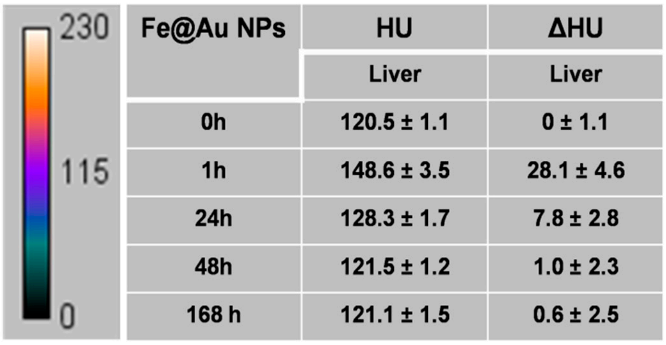


| Fe@Au NPs | HU | ΔHU |
|---|---|---|
| | Liver | Liver |
| 0h | 120.5 ± 1.1 | 0 ± 1.1 |
| 1h | 148.6 ± 3.5 | 28.1 ± 4.6 |
| 24h | 128.3 ± 1.7 | 7.8 ± 2.8 |
| 48h | 121.5 ± 1.2 | 1.0 ± 2.3 |
| 168 h | 121.1 ± 1.5 | 0.6 ± 2.5 |

**Figure S13.** Representative in vivo CT images at different experimental times (0, 1, 24, 48, and 168 h) after the intravenous injection of Fe@Au NPs. The liver has been highlighted for better observation. The table shows average HU ± SD values and differences compared to 0 h.

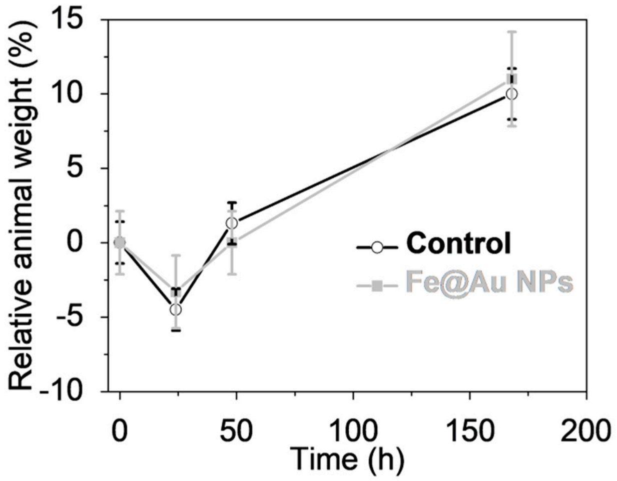


**Figure S14.** Relative weight profile of mice intravenously injected with PBS (black) and Fe@Au NPs (grey).

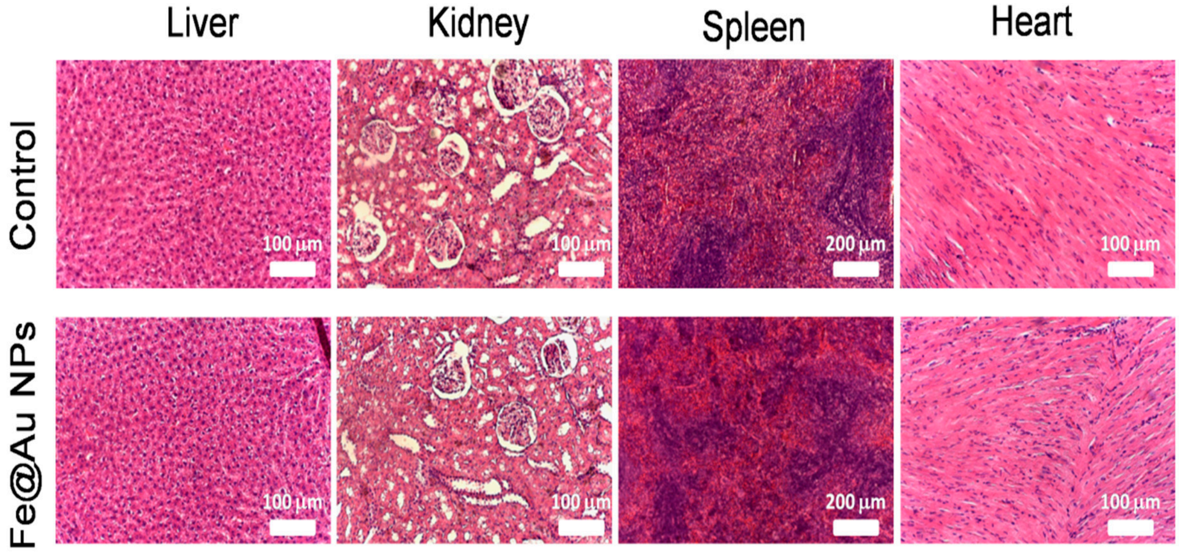


**Figure S15.** H&E staining of representative histological sections of liver, kidneys, spleen, and heart. Control mice, after 24 h post-administration of PBS (top) and 24 h post intravenous administration of Fe@Au NPs (bottom).

## References


1. Lattuada, M.; Hatton, T.A. Functionalization of monodisperse magnetic nanoparticles. *Langmuir* **2007**, *23*, 2158–2168, doi:10.1021/la062092x.
2. Barbosa, S.; Agrawal, A.; Rodriguez-Lorenzo, L.; Pastoriza-Santos, I.; Alvarez-Puebla, R.A.; Kornowski, A.; Weller, H.; Liz-Marzan, L.M. Tuning size and sensing properties in colloidal gold nanostars. *Langmuir* **2010**, *26*, 14943–14950, doi:10.1021/la102559e.
